# RND Pump-Mediated Efflux of Amotosalen, a Compound Used in Pathogen Inactivation Technology to Enhance Safety of Blood Transfusion Products, May Compromise Its Gram-Negative Anti-Bacterial Activity

**DOI:** 10.1128/msphere.00673-22

**Published:** 2023-02-28

**Authors:** Alex B. Green, Lucius Chiaraviglio, Katherine A. Truelson, Katelyn E. Zulauf, Meng Cui, Zhemin Zhang, Matthew P. Ware, Willy A. Flegel, Richard L. Haspel, Edward W. Yu, James E. Kirby

**Affiliations:** a Department of Pathology, Beth Israel Deaconess Medical Center, Boston, Massachusetts, USA; b Harvard Medical School, Boston, Massachusetts, USA; c Department of Pharmaceutical Sciences, School of Pharmacy, Bouvé College of Health Sciences, Northeastern University, Boston, Massachusetts, USA; d Department of Pharmacology, Case Western Reserve University Medical Center, Cleveland, Ohio, USA; e Department of Transfusion Medicine, NIH Clinical Center, National Institutes of Health Bethesda, Maryland, USA; Antimicrobial Development Specialists, LLC

**Keywords:** *Acinetobacter baumannii*, *Eshcerichia coli*, *Pseudomonas*, RND, adeABC, amotosalen, antimicrobial resistance, efflux, mexXY, multidrug resistance, pathogen inactivation, psoralen

## Abstract

Pathogen inactivation is a strategy to improve the safety of transfusion products. The only pathogen reduction technology for blood products currently approved in the US utilizes a psoralen compound, called amotosalen, in combination with UVA light to inactivate bacteria, viruses, and protozoa. Psoralens have structural similarity to bacterial multidrug efflux pump substrates. As these efflux pumps are often overexpressed in multidrug-resistant pathogens, we tested whether contemporary drug-resistant pathogens might show resistance to amotosalen and other psoralens based on multidrug efflux mechanisms through genetic, biophysical, and molecular modeling analysis. The main efflux systems in *Enterobacterales*, Acinetobacter baumannii, and Pseudomonas aeruginosa are tripartite resistance-nodulation-cell division (RND) systems, which span the inner and outer membranes of Gram-negative pathogens, and expel antibiotics from the bacterial cytoplasm into the extracellular space. We provide evidence that amotosalen is an efflux substrate for the E. coli AcrAB, Acinetobacter baumannii AdeABC, and P. aeruginosa MexXY RND efflux pumps. Furthermore, we show that the MICs for contemporary Gram-negative bacterial isolates for these species and others *in vitro* approached and exceeded the concentration of amotosalen used in the approved platelet and plasma inactivation procedures. These findings suggest that otherwise safe and effective inactivation methods should be further studied to identify possible gaps in their ability to inactivate contemporary, multidrug-resistant bacterial pathogens.

**IMPORTANCE** Pathogen inactivation is a strategy to enhance the safety of transfused blood products. We identify the compound, amotosalen, widely used for pathogen inactivation, as a bacterial multidrug efflux substrate. Specifically, experiments suggest that amotosalen is pumped out of bacteria by major efflux pumps in E. coli, Acinetobacter baumannii, and Pseudomonas aeruginosa. Such efflux pumps are often overexpressed in multidrug-resistant pathogens. Importantly, the MICs for contemporary multidrug-resistant *Enterobacterales*, Acinetobacter baumannii, Pseudomonas aeruginosa, *Burkholderia* spp., and Stenotrophomonas maltophilia isolates approached or exceeded the amotosalen concentration used in approved platelet and plasma inactivation procedures, potentially as a result of efflux pump activity. Although there are important differences in methodology between our experiments and blood product pathogen inactivation, these findings suggest that otherwise safe and effective inactivation methods should be further studied to identify possible gaps in their ability to inactivate contemporary, multidrug-resistant bacterial pathogens.

## INTRODUCTION

Bacterial contamination of transfusion products is currently the primary transfusion-related infectious risk (1–3), and is a leading cause of transfusion-related deaths in the United States. In particular, the need for room temperature storage of platelets allows contaminating bacteria to multiply to dangerous levels. Culture-confirmed sepsis is estimated to occur in a least 1 in 100,000 platelet transfusions without use of preemptive pathogen reduction technology (4–6).

The majority of bacterial platelet contaminants are Gram-positive skin flora. However, recent events highlight the importance of Gram-negative pathogens in transfusion-associated sepsis. In 2019, seven geographically distributed cases of sepsis resulted from platelets products contaminated with Acinetobacter calcoaceticus*-baumannii complex* isolates (ACBC). One of these occurred, despite use of pathogen reduction technology (7). A summary of blood product contaminants in the United States, the United Kingdom and France published in 2005 found that ~ 33% of platelet transfusion-associated infections were caused by either *Enterobacterales* or Acinetobacter, while the percentage of transfusion-associated infections in red blood cell products caused by these pathogens was even higher at 55% (7, 8). In addition, a recent study from the American Red Cross found that Klebsiella and Acinetobacter spp. were the Gram-negative pathogens most frequently associated with platelet transfusion-associated sepsis (6).

Accordingly, several methods for preemptive pathogen reduction in transfusion products have been developed. In particular, psoralen compounds have compelling attributes for use in pathogen reduction technologies (9–12). Psoralens are tricyclic, planar compounds capable of forming irreversible, covalent adducts with nucleic acids following excitation with long-wave UV light (i.e., UVA) (13, 14). Therefore, psoralens can be added to blood products, which are then UVA irradiated to render pathogens nonviable.

Psoralens vary in their inactivation properties. For instance, while 8-methoxypsoralen (8-MOP) is effective in inactivating many bacterial species, it is less so in inactivating viruses (15). On the other hand, while 4’-aminomethyl-4,5′,8-trimethylpsoralen (AMT) efficiently inactivates both bacterial and viral pathogens, it exhibits high mutagenicity in the Ames test, a surrogate for carcinogenic potential (15, 16). Through a medicinal chemistry effort, 4′-(4-amino-2-oxa)butyl-4,5′,8-trimethylpsoralen (also known as amotosalen) was identified, which combined 8-MOP’s safety profile with AMT’s broad spectrum efficacy (15–17). In 2014, the Cerus Corporation’s INTERCEPT Blood System, using amotosalen in conjunction with a specialized UVA illuminator, was approved by the FDA for pathogen reduction in platelets (18), and later approved for pathogen reduction in plasma (19).

Since amotosalen’s development, multiple studies have reported its efficacy against a broad spectrum of microbial pathogens (20–26). However, the existing literature, patents, and Intercept package insert only cite testing of bacterial pathogens isolated 2 decades ago when antimicrobial resistance was less common (25, 27–29). Notably, the tricyclic, planar structure of psoralens, including amotosalen, is reminiscent of known bacterial multidrug efflux pump substrates (30). Furthermore, enhanced susceptibility of an Escherichia coli *acrA* mutant to 8-MOP plus UV irradiation was previously noted in 1982 (31). It was subsequently discovered that AcrA is a critical component of the major efflux system in E. coli (32), further implicating psoralens as efflux pump substrates. Therefore, with the dramatic emergence of antimicrobial resistance, often associated with overexpression of such efflux pumps (33), we investigate here; (i) the relative susceptibility of contemporary multidrug-resistant Gram-negative pathogens to amotosalen, and (ii) whether amotosalen is a substrate for major Gram-negative efflux pumps.

## RESULTS

Assessment of amotosalen activity against contemporary multidrug-resistant E. coli, K. pneumoniae, A. baumannii, Pseudomonas aeruginosa, *Strenotrophomonas maltophilia*, and *Burkholderia* isolates was performed using MIC testing (Table 1 and Table S1). Notably, MIC values were near, and for some isolates, exceeded the 150 μM concentration of amotosalen used in the Intercept pathogen inactivation system. The modal MICs of multidrug-resistant (MDR) E. coli and K. pneumoniae (128 μM) isolates exceeded the MICs of broadly-susceptible ATCC strains of the same species (8 μM); however, broadly-susceptible A. baumannii 17978 (34) had an MIC of 128 μM, identical to the modal MIC of MDR A. baumannii isolates. Pseudomonas aeruginosa strains generally had either high MICs or were variably killed directly by UV light (Table 1 and data not shown).

**TABLE 1:** MIC for amotosalen tested against multidrug-resistant Gram-negative isolates and controls

Species	Strain #	Amotosalen modal MIC (μM)^*a*^	MDR^*b*^
A. baumannii	WRAIR 15067	128	Yes
A. baumannii	WRAIR 55	128	Yes
A. baumannii	WRAIR 846	128	Yes
A. baumannii	WRAIR 847	128	Yes
A. baumannii	WRAIR 865	128	Yes
A. baumannii	WRAIR 881	32, 64, 128	Yes
A. baumannii	WRAIR 890	128	Yes
A. baumannii	WRAIR 899	128	Yes
A. baumannii	FDA-CDC 0292	64	Yes
A. baumannii	FDA-CDC 0293	64	Yes
A. baumannii	FDA-CDC 0298	128	Yes
A. baumannii	FDA-CDC-0299	128	Yes
A. baumannii	FDA-CDC 0305	128	Yes
A. baumannii	FDA-CDC 0306	128, 256	Yes
A. baumannii	FDA-CDC 0309	64, 128	Yes
A. baumannii	FDA-CDC 0310	128	Yes
A. baumannii	ATCC^*c*^ BAA-1710 (AYE)	128, 256	Yes
A. baumannii	ATCC 17978	128	No
K. pneumoniae	FDA-CDC 0112	128, 256	Yes
K. pneumoniae	FDA-CDC 0113	128	Yes
K. pneumoniae	FDA-CDC 0120	128, 256	Yes
K. pneumoniae	FDA-CDC 0126	128	Yes
K. pneumoniae	FDA-CDC 0135	128	Yes
K. pneumoniae	FDA-CDC 0138	128	Yes
K. pneumoniae	FDA-CDC 0146	256	Yes
K. pneumoniae	FDA-CDC 0148	256	Yes
K. pneumoniae	ATCC 13883	32, 64	No
E. coli	FDA-CDC 0114	128	Yes
E. coli	FDA-CDC 0118	64	Yes
E. coli	FDA-CDC 0119	64	Yes
E. coli	FDA-CDC 0128	128	Yes
E. coli	FDA-CDC 0137	128	Yes
E. coli	FDA-CDC 0149	128	Yes
E. coli	FDA-CDC 0150	128, 256	Yes
E. coli	FDA-CDC 0151	128	Yes
E. coli	ATCC 25922	8	No
P. aeruginosa	ATCC 27583	<4	No
P. aeruginosa	FDA-CDC 229	>256	Yes
P. aeruginosa	FDA-CDC 230	128	Yes
P. aeruginosa	FDA-CDC 231	>256	Yes
P. aeruginosa	FDA-CDC 232	256	Yes
Burkholderia cenocepacia	BEI^*d*^ NR-701	128	Unknown
Burkholderia cenocepacia	BEI NR-20535	256	Unknown
Burkholderia cepacia	BEI NR-702	>256	Unknown
Burkholderia cepacia	BEI NR-707	Variable	Unknown
Burkholderia multivorans	BEI NR-20532	Variable	Unknown
Burkholderia multivorans	BEI NR-20533	>256	Unknown
Burkholderia multivorans	BEI NR-20534	>256	Unknown
Burkholderia stabilis	BEI NR-706	0	Unknown
Stenotrophomonas maltophilia	JEK154	256	
Stenotrophomonas maltophilia	JEK155	64	

a Amotosalen MIC data were determined experimentally as described in materials and methods. Modal MIC values are from 3 or more experimental replicates. A concentration of zero for modal MIC indicates complete growth inhibition was observed following UVA exposure in the absence of amotosalen treatment. Variable = MIC range spanning at least 4 doubling dilutions with no modal MIC. S. maltophilia strains were randomly chosen clinical isolates from the Beth Israel Deaconess Medical Center.

b Definition of multidrug resistance (MDR) was from Margiorakos et al. (95). Specifically, MDR was assigned if there were resistance to 1 or more antimicrobials in 3 or more antimicrobial categories (aminoglycosides, fluoroquinolones, carbapenems, etc.). In this classification, extensively drug-resistant (XDR) is designated if there were resistance to one or more antimicrobials in all but 2 or fewer antimicrobial classes. Pandrug-resistant (PDR) is designated if there were resistance to all antimicrobials. In our study, assignment of XDR and PDR categorization was not possible based on absence of complete susceptibility profiling for most of the strains examined. Extended susceptibility data for isolates is provided in Table S1 where available.

c ATCC = American Type Culture Collection (Manassas, VA).

d BEI = BEI Resources (Manassas, VA).

10.1128/msphere.00673-22.1 Table S1 Modal minimal inhibitory concentrations (MIC) for amotosalen and susceptibility to other antimicrobials for individual bacterial isolates examined. Download Table S1, XLS file, 0.06 MB. Copyright © 2023 Green et al. 2023 Green et al. https://creativecommons.org/licenses/by/4.0/ This content is distributed under the terms of the Creative Commons Attribution 4.0 International license.

We then examined whether well-characterized, major efflux pumps from E. coli, A. baumannii and P. aeruginosa were capable of influencing susceptibility to psoralen compounds. These pumps are classified as resistance-nodulation-cell division (RND) efflux pumps, and consist of 3 components residing, respectively, in the inner membrane, periplasm, and outer membrane.

In E. coli, multiple RND efflux pumps depend on a shared outer membrane channel protein, TolC (35). We, therefore, compared psoralen MICs in an E. coli K-12 parent strain and a TolC-knockout (Δ*tolC*) strain that were otherwise genetically identical (i.e., isogenic strains). For amotosalen, 8-MOP and AMT (Table 2), the MIC in the Δ*tolC* background was reduced by up to 64-fold, consistent with TolC-dependent efflux of psoralens. The magnitude of MIC reduction was similar to that observed for positive controls, clindamycin, fusidic acid, ethidium bromide, and minocycline, known efflux substrates (33, 36, 37). As expected, based on prior observations (36), non-effluxed, gentamicin and apramycin aminoglycosides, and meropenem; and poorly effluxed, rifampin negative controls showed minimal or no TolC-dependent change in MIC (38–40). Furthermore, phenylalanine-arginine β-naphthylamide (PAβN), a competitive inhibitor of RND efflux pumps (41), reduced the MICs of psoralens including amotosalen in the wild-type strain by approximately 4-fold, but without significant additional effect on the *tolC* mutant, also supporting a major contribution of RND efflux pumps to observed psoralen resistance in the E. coli K-12 strain background.

**TABLE 2:** Effect of a Δ*tolC* deletion and the efflux pump inhibitor, PAβN, on the minimal inhibitor concentration of amotosalen and other psoralens in E. coli K-12^*a*^

Antimicrobial	wt	Δ*tolC*	wt + PAβN	Δ*tolC* PAβN
Amotosalen	128	2	32	4
8-MOP	>256	`32	128	32
AMT	64	8	16	8
CLI^P^	64	2	4	2
MIN^P^	16	2	N.D.	N.D.
EtBr^P^	512	16	N.D.	N.D.
FA^P^	>128	2	>128	0.5
CHL^P^	8	2	4	2
APR^N^	4	4	8	4
GEN^N^	0.5	0.25	0.5	0.5
MER^N^	0.125	0.25	N.D.	N.D.
RIF^N^	4	4	1	1

Escherichia coli Keio wild type (wt) strain and isogenic Δ*tolC* mutant, that cannot produce functional RND (resistance-nodulation-division) efflux transporters due to the absence of the required TolC outer membrane component, were used for analysis. Modal MIC (MIC) was determined from three technical replicates and are representative of at least two separate experiments, listed in μM for amotosalen and μg/mL for other antibiotics. PAβN = phenylalanine-arginine β-naphthylamide at 18 μM (6.75 μg/mL).

a 8-MOP, 8-methoxypsoralen; AMT, 4’-aminomethyltrioxsalen hydrochloride; CLI, clindamycin; MIN, minocycline; EtBr, ethidium bromide; FA, fusidic acid; CHL, chloramphenicol; APR, apramycin; GEN, gentamicin; MER, meropenem; RIF, rifampin; Superscripts following drug names; Superscript P, positive control; Superscript N, negative control; N.D., not done.

In A. baumannii and other Acinetobacter species, multidrug resistance is commonly associated with upregulation of the AdeABC RND efflux system (42–44). To examine whether AdeABC could efflux amotosalen, we cloned *adeAB* and *adeC* on separate plasmids under the control of inducible promoters. These plasmids were then transformed alone or in combination into the E. coli AG100AX strain, which is deleted for the main RND efflux pumps (Δ*acrAB*, Δ*acrEF*) that partner with TolC, thereby removing confounding effects of native efflux pumps in the host strain (45).

For amotosalen, the modal MIC of the AdeC expressing control strain, i.e., an incomplete, inoperative pump, was found to be 8 μM, which was comparable with the modal MIC of the AG100AX parent E. coli strain (Table 3). In contrast, a strain expressing an induced, intact AdeABC pump, exhibited a 32-fold higher modal MIC of 256 μM. Therefore, psoralen MIC was substantially increased in the presence of the complete heterologous AdeABC RND pump, providing evidence for efflux of amotosalen by this alternative system. Similarly, expression of *adeABC* increased the MIC of the known efflux substrate, minocycline, approximately 10-fold, while having relatively little effect on rifampin, a substrate of a different pump in A. baumannii, *adeIJK* (46), and apramycin, a poor substrate and therefore a negative control. Lastly, we noted that expression of E. coli *acrAB* and P. aeruginosa *mexXY* in AG100AX led to a substantial increase in amotosalen MIC, while expression of the A. baumannii AdeIJK and P. aeruginosa MexAB pumps or a vector expressing an unrelated fluorescent protein used as a negative control had negligible, if any, effect (Table 4). In these experiments, minocycline MIC was modestly increased in the AdeIJK and MexXY strains as expected (32, 47). Surprisingly, an increase in gentamicin MIC was minimal, or not observed, with expression of MexXY, a pump that is responsible for efflux of aminoglycosides in P. aeruginosa. This was the case even when the outer membrane protein, OprM, believed to partner with MexXY in P. aeruginosa, was expressed from a separate plasmid in a Δ*tolC* deletion strain. In contrast, the MIC of amotosalen was increased 64-fold, when MexXY and OprM were co-expressed in this strain background (data not shown).

**TABLE 3:** Effect of a heterologously expressed AdeABC pumps from A. baumannii on amotosalen MIC^*a*^

Antimicrobial	pAdeC	pAdeAB + pAdeC
Amotosalen	8	256
RIF	4	8
MIN	0.06	0.5
APR	4	4

a Pumps were expressed in E. coli strain AG100AX lacking major endogenous E. coli RND efflux pumps (acrABacrEF). The tripartite A. baumannii AdeABC pump was expressed on 2 plasmids encoding AdeAB and AdeC, respectively. The pump is only active when both plasmids are present in the same strain. Values shown are modal MICs, representative of at least 2 separate experiments with 3 to 4 technical replicates per experiment.

**TABLE 4:** Effect of heterologously expressed AcrAB from E. coli, AdeIJK from A. baumannii, and MexAB-OprM and MexXY from P. aeruginosa on amotosalen MIC^*a*^

Antimicrobial	pBad-LSS-Orange control	pBad-*adeIJK*	pBad-*acrAB*	pBad-*mexAB-oprM*	pBad-*mexXY*
Amotosalen	8	16	256	8	128
MIN	0.5	0.25	2	0.5	2
GEN	0.25	0.25	0.25	0.25	0.25

a Pumps were expressed in E. coli strain AG100AX lacking major endogenous E. coli RND efflux pumps (acrABacrEF). The A. baumannii AdeIJK, E. coli AcrAB, or P. aeruginosa MexAB-OprM or MexXY efflux pumps were expressed as a single operon under inducing conditions. pBAD-LSS-Orange is a vector control expressing an unrelated fluorescent protein in the same vector background used for expression of the RND efflux operons indicated. Values shown are modal MICs, representative of at least 2 separate experiments with 3 to 4 technical replicates per experiment.

Traditionally, MIC testing is performed in the absence of serum proteins; however, such testing may overestimate activity if lower amounts of free drug are available for antimicrobial activity due to binding to circulating proteins, such as albumin. Therefore, as psoralens are relatively hydrophobic, we considered the potential effect of human serum binding on its activity. We performed experiments in the Δ*tolC* E. coli K-12 strain background to allow us to evaluate the extent of MIC increase within the testable range of amotosalen concentrations. However, E. coli K-12 was inhibited by 50% serum. It has been observed previously that many strains do not grow in the presence of serum (48), potentially necessitating the use of alternatives, such as human serum albumin (HSA) as a surrogate or adaptation of strains to serum containing medium. We found, however, that addition of 1 mg/mL of sodium polyanethole sulfonate (SPS), a standard additive in blood culture medium, alleviated observed serum inhibition, which is consistent with its known ability to block complement- and non-complement-mediated serum bactericidal activity (49). Notably, MIC of amotosalen was not consistently increased in the presence of serum, but increased 16-fold with 2% HSA (Table 5). Therefore, there were discordant effects of serum and HSA on amotosalen activity. Fusidic acid, protein binding of 97 to 99% (https://go.drugbank.com/drugs/DB02703), was used a positive control, and showed a similar 8- to 16-fold increase in MIC in the presence of either serum or HSA. In contrast, chloramphenicol with a plasma protein binding of roughly 50% (https://go.drugbank.com/drugs/DB00446) showed a much smaller increase in MIC, while the activity of apramycin, observed to have < 10% serum protein binding in a recent study (50), was modestly enhanced in the presence of serum or HSA. It should be noted that the observations for serum and HSA may differ from potential effects of plasma present in blood products on amotosalen during the pathogen inactivation procedure.

**TABLE 5:** Effect of human serum and albumin on amotosalen activity in E. coli K-12 Δ*tolC* background^*a*^

Antimicrobial	No additive	SPS^*b*^	SPS + 50% serum	2% HSA^*c*^
Amotosalen	2	4	4	32
FA^*d*^	2	2	32	32
CHL^*e*^	1	0.5	0.5	1
APR^*f*^	4	8	2	1

a MIC listed in μg/mL for FA, CHL, and APR, apramycin. Data are modal MICs values for 3 technical replicates and are representative of at least 2 separate experiments.

b SPS, sodium polyanethole sulfonate.

c HSA, human serum albumin.

d FA, fusidic acid.

e CHL, chloramphenicol.

f APR, apramycin.

Transposon mutants in RND pumps in widely researched strains of A. baumannii AB5075 (51, 52) and P. aeruginosa PAO1 (53) from the University of Washington transposon bank were also examined for effects on amotosalen MIC (Table 6). Independent mutants in *adeB* showed a 4-fold lower MIC, while mutants in other A. baumannii and P. aeruginosa pumps examined did not result in an observable change, although it should be noted that the baseline level of resistance in the P. aeruginosa PAO1 strain background was beyond the measurement range of our assay.

**TABLE 6:** Effect of an isogenic RND efflux pump mutants in A. baumannii *and* P. aeruginosa on minimal inhibitor concentration of amotosalen

Species	Strain	Genotype	MIC^*a*^ (μM)
A. baumannii	AB5075-UW	Wild type	256
A. baumannii	AB05228	*adeB*	64
A. baumannii	AB05230	*adeB*	64
A. baumannii	AB02295	*adeJ*	256
A. baumannii	AB02296	*adeJ*	256
A. baumannii	AB02297	*adeJ*	256
A. baumannii	AB03594	*adeG*	256
A. baumannii	AB03595	*adeG*	256
A. baumannii	AB03596	*adeG*	256
P. aeruginosa	MP01	Wild type	>256
P. aeruginosa	PW1778	*mexA*	>256
P. aeruginosa	PW1779	*mexA*	>256
P. aeruginosa	PW1780	*mexB*	>256
P. aeruginosa	PW1781	*mexB*	>256
P. aeruginosa	PW4498	*mexY*	>256
P. aeruginosa	PW4499	*mexY*	>256

a MIC, listed in μM for amotosalen.

**FIG 1:**
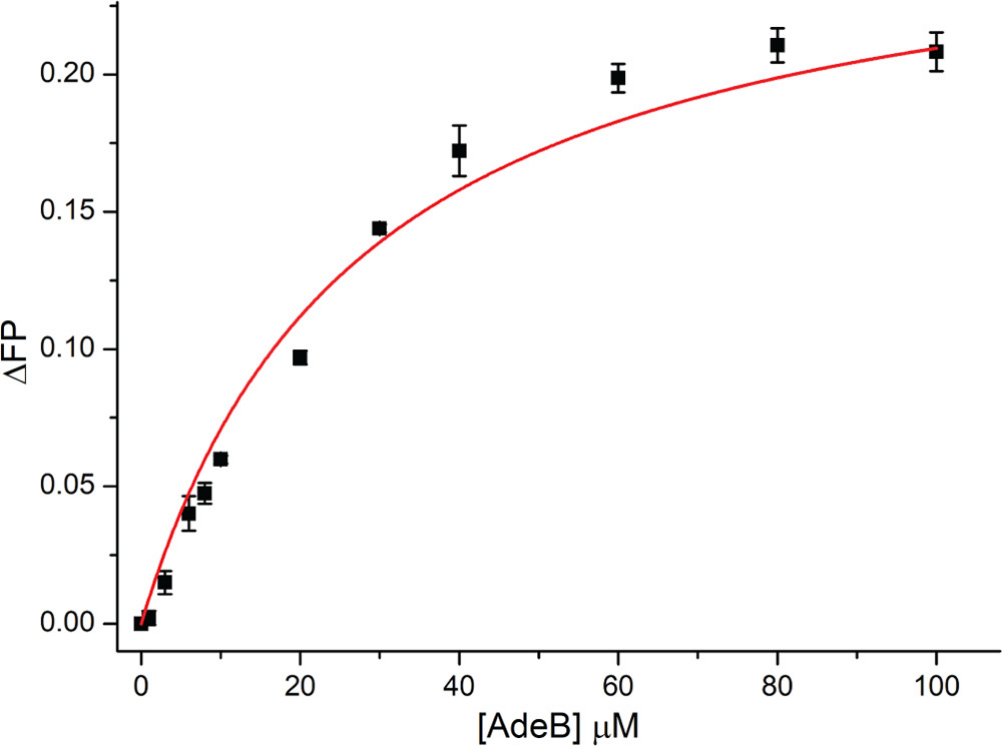
Binding affinity of amotosalen and AdeB determined using fluorescence polarization. Indicated concentrations of AdeB were mixed with 3 μM amotosalen. The change in fluorescence polarization signal (ΔFP) indicates a K_D_ of 27.9 ± 1.8 μM for amotosalen.

In the A. baumannii AdeABC efflux pump, the AdeB protein is the drug-binding channel and pump, energized by a proton motive force, which in coordination with other pump components, extrudes compounds from the bacterial cell (54). We, therefore, assessed the ability of amotosalen to bind purified AdeB using a fluorescence polarization assay, taking advantage of the inherent fluorescence of amotosalen (Fig. 1). The dissociation constant (K_D_) for binding to AdeB was 27.9 ± 1.8 μM, in the same range as the K_D_ of rhodamine 6G (3.1 μM) and ethidium bromide (2.5 μM) for AdeB (55), and the K_D_ of ethidium bromide (8.7 μM), proflavin (14.5 μM), and ciprofloxacin (74.1 μM) efflux substrates for the homologous, AcrB (30).

**FIG 2:**
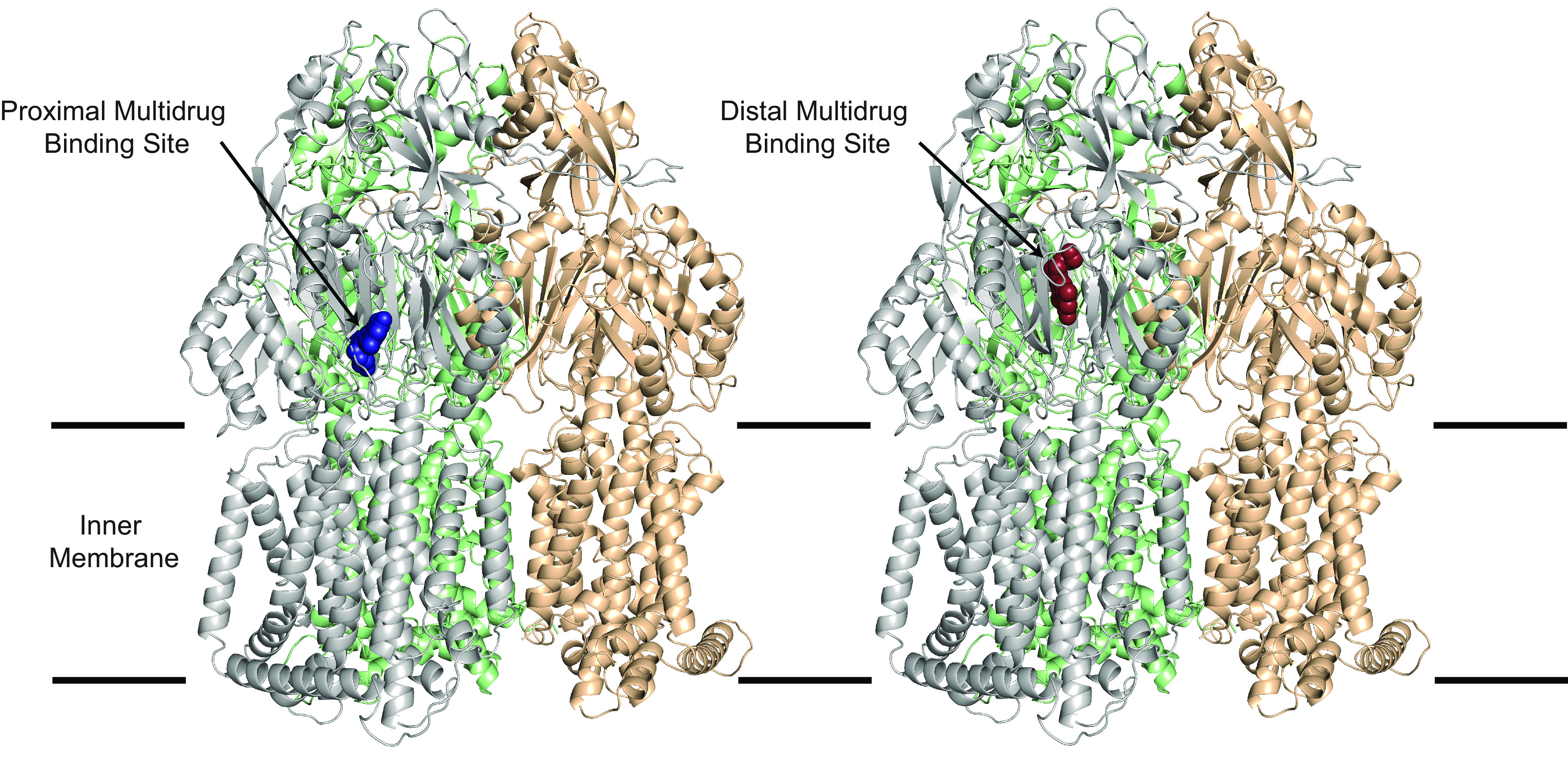
Modeling of amotosalen binding to AdeB. Two amotosalen docking sites were identified in the periplasmic domain of the recently determined cryo-EM structure of AdeB (55) using Induced Fit Docking and XP scores for ranking. The sites are within the hydrophobic cleft between PC1 and PC2 subdomains in AdeB that are thought to form the entry site and pathway for efflux from the periplasm (54). Amotosalen is predicted to bind proximal and distal drug binding sites, as previously defined through autodocking and experimental analysis of antibiotic efflux substrates for AdeB, and homologous RND transporters (54, 92, 93). Specifically, critical contacts are made with hydrophilic residue D664 in the PAIDELGT sequence defined “F-Loop,” which forms both part of the cleft entrance and the proximal drug binding site. Amotosalen also binds to the distal multidrug binding site inclusive of hydrophobic interactions with F178, I607, and W610. This hydrophobic patch in the homologous AcrB is critical for stable binding of all substrates, and is further highlighted in corresponding residue interactions in the AcrB-minocycline crystal structure and corresponding MtrD-erythromycin cryo-EM structure (54, 92–94). The predicted binding affinities (XP scores) to the 2 sites are −10.63 and −11.66, respectively.

The previously described cryo-EM structure of AdeB identified a pathway for efflux pump substrate extrusion with entrance through a cleft in the periplasmic domain, and sequential binding to proximal and distal multidrug-binding sites (54, 55). Computer modeling of the molecular docking of amotosalen using Extra Precision Glide (56) demonstrated binding to both the proximal and distal multidrug binding sites within the AdeB periplasm domain (Fig. 2). AMT and 8-MOP were also found to dock with the proximal and distal binding sites, and cleft and distal binding sites, respectively (data not shown). These data are consistent with binding of known efflux substrates to AdeB and homologous RND pumps (30).

## DISCUSSION

**FIG 3:**
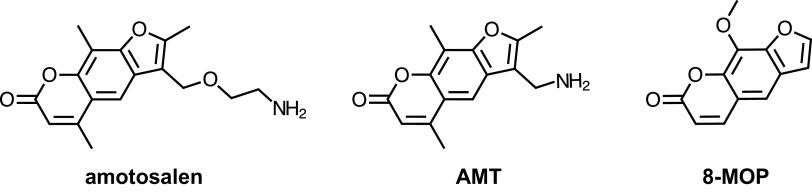
Chemical structures of amotosalen, AMT, and 8-MOP. These related psoralens share a core planar structure with few rotatable bounds. However, amotosalen and 4’-aminomethyltrioxsalen (AMT) also contain primary amino groups, which when combined with molecular planarity and small numbers of rotatable bonds, are associated with enhanced penetrance into Gram-negative pathogens (57). Therefore, structural properties appear to explain the observed enhanced activity of amotosalen and AMT, compared with 8-MOP.

Microbial contamination of blood products remains a critical transfusion safety issue. Here, we provide evidence that psoralens including amotosalen are multidrug efflux substrates. Noted, we did not directly demonstrate efflux using physicochemical methods; however, heterologous expression and gene mutation data, and the inhibitory effects of the well-established efflux pump inhibitor, PAβN, are highly supportive of an efflux mechanism. It is of interest that the derivatization of psoralen scaffold to create amotosalen and AMT included the instillation of a primary amine into an existing planar structure with low globularity and few rotatable bonds (Fig. 3). These features, taken together, are now known to be associated with enhanced penetration of antibiotics across the Gram-negative membrane barrier (57). Therefore, the increased activity (lower MIC values) of amotosalen and AMT compared with 8-MOP is fully consistent with our current understanding of antimicrobial penetrance into Gram-negative pathogens. Nevertheless, access to an intracellular target (in this case, DNA) appears especially vulnerable to efflux mechanisms.

The major RND efflux pumps that efflux amotosalen, AcrAB-TolC, AdeABC, and MexXY, have overlapping substrate specificity, and 40 to 50% amino acid identity (58, 59). Therefore, finding that these separate systems all efflux psoralens is not surprising. Interestingly, amotosalen appeared to be selectively effluxed by MexXY rather than MexAB. Previously, these 2 pumps were found to have some substrates in common and some unique substrates(59). Amotosalen now joins aminoglycosides among compounds selectively effluxed by MexXY. Genetic evidence suggested the MexXY-dependent efflux of amotosalen occurs in E. coli with either native TolC or heterologously expressed, P. aeruginosa OprM serving as outer membrane partner.

Binding of amotosalen to AdeB showed a micromolar dissociation constant, consistent with known affinities of antibiotics to RND pumps (30) and MATE family transporters (60). This affinity is likely located along a dissociation continuum that allows for the ability to bind to several sites within the pump itself with eventual handoff to the outer membrane pump protein, and release into the extracellular space (54). Molecular docking to AdeB was consistent with what has been either determined or predicted for other canonical efflux substrates (55).

AcrAB-TolC AdeABC, and MexXY are upregulated in resistant pathogens. For example, *acrAB* is upregulated in Enterobacter species resistant to colistin (61); E. coli *r*esistant to multiple antibiotics (62–64); and in drug-resistant Salmonella (65, 66) and Klebsiella (67, 68). The expression of AdeABC is often upregulated in resistant Acinetobacter isolates (42–44). MexXY is normally relatively suppressed, but when upregulated is responsible for inducible pan-aminoglycoside resistance in P. aeruginosa (69), and such strains may be even more resistant to amotosalen than those in our analysis. The lack of a substantial effect of *mexXY* mutants in P. aeruginosa PAO1, i.e., bringing the MIC into a measurable range, may reflect contributions of additional pumps (there are 12 RND pumps in PAO1) not examined in our study (70). In addition, Stenotrophomonas maltophilia, and *Burkholderia* spp. express or overexpress multiple efflux pumps, resulting in characteristic intrinsic resistance to many antibiotics (71–73).

Therefore, it is perhaps not surprising that we found relative resistance to amotosalen among MDR isolates where a significant fraction had MICs exceeding the concentration of amotosalen used in the Intercept pathogen inactivation procedure. Although we cannot, without extensive genetic and expression analysis, definitively conclude that higher MIC values across this strain set were due to efflux activity alone, it is reasonable to hypothesize that efflux was a major contributor.

It was of additional interest that we identified the addition of SPS as a method to potentially address growth inhibition during MIC testing with serum, an issue in the field (48). Clearly, these findings should be replicated with additional pathogens to determine its broad applicability; however, we do note the use of SPS as standard additive in blood culture medium that appears to alleviate growth inhibition by human blood (49). Oddly, human serum albumin, but not serum, significantly increased the amotosalen MIC. This potentially may indicate that the binding site for amotosalen in the serum tested was not accessible.

Interestingly, a minority of *Burkholderia* spp. isolates and Pseudomonas aeruginosa were either always or variably killed by UVA light in the absence of amotosalen exposure (Table 1 and data not shown). Based on prior literature, we speculate this may result from free radical generation during UVA excitation of bacterial fluorescent pigments and/or endogenous photosensitizers (74, 75).

Though generally effective against most pathogens, psoralens are ineffective against non-enveloped viruses (e.g., HAV, HEV, parvovirus B19, and poliovirus) and relatively imporous bacterial spores (76–78). Taken together, our data now raise the possibility that contemporary multidrug-resistant bacterial isolates have reduced susceptibility to psoralen inactivation based on efflux. Collectively, our data also suggest the need for further study of psoralen-efflux pump interaction, and that future chemical optimization of pathogen inactivating compounds should specifically explore Gram-negative penetrance in the presence of efflux pumps.

Our study has several limitations. Notably, we did not employ the INTERCEPT Illuminator for UVA exposure. We tested inactivation of pathogens in standard antimicrobial susceptibility testing medium, and in lid-less microwell plates with a very short path length for UVA exposure, not in blood products contained in bags with potentially greater UVA opacity, and therefore, to compensate, used a slightly lower 2J/cm^2^ dose of UVA compared to the Intercept 3J/cm^2^ dose. As such, our results may differ from inactivation achieved in the INTERCEPT system. Also, our results should be taken in context. MDR Gram-negative pathogens, thus far, are relatively rare causes of transfusion associated sepsis. Furthermore, even partial killing might be sufficient to prevent a sepsis event, even if not demonstrating full susceptibility in a presumably more stringent MIC test. Finally, pathogen inactivation strategies have provided significant benefit in reducing the overall frequency of transfusion-associated bloodstream infection (79).

Nevertheless, emerging antimicrobial resistance, including resistance associated with efflux mechanisms, is becoming increasingly common. The failure of pathogen reduction to completely eliminate contaminating pathogens depending on bacterial load has been recently reviewed (80), and we suggest that these observations may in part be related to the ability of some pathogens to efflux inactivation agents. Our findings serve as an alert to a potential vulnerability in pathogen inactivation methods that may explain some instances of pathogen inactivation breakthrough and should be an area of further research.

## MATERIALS AND METHODS

### Chemicals.

Amotosalen HCl 3 mM solution (lots CE19F14L71 and CE20J12L71) was obtained from the Cerus INTERCEPT Blood System for Platelets Pathogen Reduction System Dual Storage Processing Set and stored in light-protected aliquots at 4°C. AMT and 8-MOP, both from Sigma-Aldrich, were dissolved in ~ 4% dimethyl sulfoxide (DMSO) and 100% DMSO, respectively, and stored as aliquots at −80°C prior to use. Other antimicrobials used were apramycin (Alfa Aeser), clindamycin (Sigma-Aldrich), chloramphenicol (Sigma-Aldrich), fusidic acid (Chem-Impex International), gentamicin (Alfa Aeser), minocycline (Chem-Impex International), rifampin, and PAβN (MedChem Express).

### Bacterial strains.

Clinical bacterial isolates are listed in Table S1, and were obtained from the American Type Culture Collection (ATCC), the CDC-FDA Antimicrobial Resistance Isolate Bank (ARIB), Walter Reed Army Institute of Research (WRAIR), and BEI Resources. The *Keio* strain, BW25113, and isogenic, JW5503-KanS Δ*tolC* E. coli were obtained from the Coli Genetics Stock Center (Yale University, New Haven, CT) (81). E. coli AG100AX Δ*acrAB* Δ*acrEF* (82) was from Ed Yu (Case Western University, Cleveland, OH). We gratefully acknowledge receipt of A. baumannii AB-7075 and P. aeruginosa PAO1 transposon mutants from the Manoil Laboratory (University of Washington, Seattle, WA).

### Creation of isogenic AdeABC efflux strains.

Vectors for regulated expression of *adeAB* and *adeC* were created as follows: To create the isopropyl β-D-1-thiogalactopyranoside (IPTG)-inducible, pAdeC vector, pBMTL-2 (83) was first converted to pBMTL-2NTC by replacing the kanamycin resistance gene with a nourseothricin acetyltransferase resistance gene. Specifically, pBMTL-2 was amplified by PCR using “F pLAC (NAT)” and “R pLAC (Nat)” primers (Table S2), and the nourseothricin resistance gene was amplified from plasmid pMOD3-mNeptune2-nat (84) (Addgene #120335) using primers “F Nat” and “R Nat” with inclusion of 5′ tails encoding overlap between vector and nourseothricin amplicons, respectively. pBMTL-2 was a gift from Ryan Gill (Addgene #22812). All amplification reactions were performed using Q5 high-fidelity DNA polymerase (New England Biolabs), followed by DpnI digestion, column-purification (Qiaquick PCR Purification kit, Qiagen) assembly using the HiFi reaction kit (New England Biolabs). Transformants were selected on 50 μg/mL nourseothricin. The *adeC* gene from A. baumannii strain AYE (ATCC BAA-1710) was then similarly amplified from genomic DNA using primers “F AdeC” and “R AdeC,” and cloned downstream from the vector pLac site and Shine-Delgarno sequence in pBMTL-2NTC using vector primers, “F pLAC (AdeC)” and “R pLAC (AdeC),” with the new vector, again, assembled using HiFi as described above.

10.1128/msphere.00673-22.2 TABLE S2 Primers for RND efflux pump vector constructs. Download Table S2, DOCX file, 0.02 MB. Copyright © 2023 Green et al. 2023 Green et al. https://creativecommons.org/licenses/by/4.0/ This content is distributed under the terms of the Creative Commons Attribution 4.0 International license.

To create the arabinose-inducible pAdeAB vector, pBAD-LSSmOrange (85) was amplified using primers “R pBAD” and “F pBAD” to exclude the existing fluorescent protein. pBAD-LSSmOrange was a gift from Vladislav Verkhusha (Addgene plasmid # 37129). *adeAB* genes were amplified from A. baumannii strain AYE genomic DNA, while adding a Shine-Dalgarno sequence using primers “F AdeA” and “R AdeB”. Amplicons were assembled by HiFi.

Similarly, the respective primer sets listed in Table S2 were used to PCR amplify the remaining RND pumps from E. coli BW25113, A. baumannii AYE, P. aeruginosa PAO1, and P. aeruginosa 27853, which were cloned using HiFi into pBAD-LSSmOrange (85) to create pAcrAB, pAdeIJK, pMexAB-OprM, and pMexXY vectors, respectively. P. aeruginosa OprM was similarly cloned into pBMTL-3 (Addgene #22813) (83). All plasmid constructs were confirmed by Sanger sequencing and introduced into a chemically-competent E. coli strain using the 1X TSS method (86).

### MIC determination.

For MIC determination, bacterial stocks frozen at −80°C were streaked onto tryptic soy agar plates containing 5% sheep blood (Remel), and grown overnight at 35°C in ambient air. Colonies were then suspended in 0.9% saline to 0.5 McFarland, measured using a Densicheck (bioMérieux); diluted 1:300 in cation-adjusted Mueller-Hinton broth (BD); and dispensed into 384-well polystyrene plates (Greiner Bio-One) at 50 μL per well with the following exceptions: For MIC determinations with AG100AX strains containing pAdeC and pAdeAB plasmids, −80°C frozen stocks were inoculated directly into non-cation-adjusted Mueller-Hinton broth containing 100 μg/mL ampicillin, 50 μg/mL nourseothricin, 5 mM calcium chloride, and 5 mM magnesium chloride, with or without *adeABC* induction using 1% L-arabinose and 1.0 mM Isopropyl β-D-1-thiogalactopyranoside (IPTG), and grown overnight at 37°C in 15 mL conical tubes with continual rotation. Bacterial cultures were then adjusted to 0.5 McFarland and diluted 1:300 in the same medium, and dispensed as above. Induction of other cloned efflux pumps was with 1% L-arabinose (or 0.5% L-arabinose for cloned P. aeruginosa pumps) in the absence of supplemental divalent cations. Lastly, P. aeruginosa PAO1 strains were dispensed into 96-well plates to avoid skipped-well effects observed in 384-well plate format.

Filled microplates were centrifuged at 1250 rcf for 4 min to ensure the entire inoculum was in continuity with the bottom of the well. Then, 2-fold doubling dilutions of stock solutions of amotosalen, AMT, or 8-MOP supplemented with 0.3% Tween 20 (Sigma-Aldrich) were dispensed into microwells using the HP D300 digital dispensing system (HP, Inc.), as previously described (87–90), and the microplates were mixed for 5 min on a microplate shaker to ensure complete mixing of psoralens with the inoculum. Microplates were then exposed to UV light in a UV Stratalinker 1800 (Stratagene), retrofitted with UV BL F8T5 CFL 12-inch UVA 365 nm Blacklight Bulbs (Coolspider), and calibrated according to manufacturer’s instructions with an UVA365 UV Light Meter (Amtast) for a total exposure of 2 Joules/cm^2^ of UVA, to approximate exposure during platelet pathogen inactivation (10). Testing of control antibiotics was performed in an identical fashion, however, without UVA exposure.

After incubation at 35°C for 20 h, the A_600_ was measured using a TECAN M1000 microplate reader. MICs were determined based on a growth inhibition A_600_ cutoff of 0.06, which is consistent with our prior determinations of Clinical and Laboratory Standards Institute reference standard equivalent, MIC determinations in 384-well plate format (88, 90, 91). Notably, the AdeABC pump was inactive in E. coli AG100X in Mueller-Hinton broth without the addition of divalent cations, consistent with prior use of magnesium supplementation when expressing this A. baumannii efflux pump in E. coli (32). The concentrations of MgCl_2_ and CaCl_2_ used were empirically determined previously to be optimal for efflux of minocycline and ethidium bromide (data not shown).

### AdeB binding affinity and molecular docking.

His-tagged AdeB protein was purified as described previously (30). Fluorescence polarization assays were performed in a ligand binding solution consisting of 20 mM HEPES-NaOH pH 7.5 and 0.05% n-dodecyl-β-d-maltoside (DDM) and 3 μM amotosalen. The experiments were done by titrating the AdeB protein in solution containing 20 mM HEPES-NaOH pH 7.5 and 0.05% DDM into the ligand binding solution while keeping DDM concentration constant. Fluorescent polarization was measured at 25°C using a PerkinElmer LS55 spectrofluorometer, coupled with a Hamamatsu R928 photomultiplier. The excitation wavelength for amotosalen was 350 nm and the fluorescent polarization signal (ΔP) was measured at 470 nm. Titration data points represent 15 measurements, and 3 biological replicates were performed to determine the K_D_ as previously described (30). ORIGIN Version 7.5 (OriginLab Corp.) was used for curve fitting.

### Molecular docking.

A structure of the “binding” protomer of AdeB-Et-I (PDB ID: 7KGH) was used as the template, in which the bound Et ligands were removed from the protomer (55). Induced-fit-docking was performed using Extra Precision Glide (56) with default parameters in the Protein Preparation Wizard Module of Maestro (Release 2019-3) (Schrödinger) to predict binding modes of amotosalen, AMT, and 8-MOP to AdeB. For each calculation, residues within 5 Å of the bound ligand were selected for side chain optimization using Prime refinement. The docking results with the lowest XP scores were selected as predicted poses.
