# Supplementary material for: RND Pump-Mediated Efflux of Amotosalen, a Compound Used in Pathogen Inactivation Technology to Enhance Safety of Blood Transfusion Products, May Compromise Its Gram-Negative Anti-Bacterial Activity
Source: mSphere. 2023 Feb 28;8(2):e00673-22. doi: 10.1128/msphere.00673-22 (PMC10117049; doi:10.1128/msphere.00673-22)
Supplement: TABLE S2 [file msphere.00673-22-s0002.docx]

**Table S2. Primers for RND efflux pump vector constructs.**

| F pLAC (NAT) | gagcatgccctgcccgtaagggcggatccccctca |
| --- | --- |
| R pLAC (NAT) | tgtcatcaagggtcgtacccattttagcttccttagctcctgaaaatctcgataactc |
| F NAT | gctaaggaagctaaaATGGGTACGACCCTTGATGACACG |
| R NAT | cttgagggggatccgcccTTACGGGCAGGGCATGCTCATG |
| F pLAC (AdeC) | ggaatatcaaaagtctaacagactccagcgtaactggactg |
| R pLAC (AdeC) | cattttttttcctccttctcaagcttgaattcgttgacgaattctctagag |
| F AdeC | caagcttgagtaaggaggaaaaaaaaTGGAGAATACTATGTCTAAATCGGCAATCG |
| R Ade C | ttacgctggagtctgTTAGACTTTTGATATTCCTCCTCCTAAAACTTTATAGAGTTC |
| F pBAD | aaatctcatcctaagaattcgaagcttggctgttttggcg |
| R pBAD | GATGCTTTTGCATACTGTCCATGGTTAATTCCTCCTGTTAGCCCAAAAAAC |
| F AdeA | aggaggaattaaccATGGACAGTATGCAAAAGCATCTTTTACTTC |
| R AdeB | ccaagcttcgaattcTTAGGATGAGATTTTTTTCTTAGAGGAAAATAGCTTTTC |
| F AdeB | ATGATGTCACAATTTTTTATTCGTCGTCC |
| R AdeA | CGACGAATAAAAAATTGTGACATCATGGTTGc |
| F pBAD (adeIJK hom) | AAAAAGCAATAATAAgaattcgaagcttggctgttttg |
| R pBAD (adeIJK hom) | CCGACATCATggttaattcctcctgttagccca |
| F adeIJK (pBAD hom) 1 | ggctaacaggaggaattaaccATGATGTCGGCTAAGCTTTG |
| R adeIJK (pBAD hom) 1 | cagccaagcttcgaattcTTATTATTGCTTTTTAAGTTCAGCACTAGATG |
| F pBAD (acrAB hom) | atactgtcgatcatcattgatTAAgaattcgaagcttggctgttttgg |
| R pBAD (acrAB hom) | aaccctctgtttttgttcatggttaattcctcctgttagccc |
| F acrAB (pBAD hom) | ctaacaggaggaattaaccatgaacaaaaacagagggtttacgcc |
| R acrAB (pBAD hom) | gcttcgaattcTTAatcaatgatgatcgacagtatggctgtgctcg |
| F pBAD (mexA-oprM hom) | gatccccaggcttgaTAAgaattcgaagcttggctgttttg |
| R pBAD (mexA-oprM hom) | tggcgttcgttgcatggttaattcctcctgttagccca |
| F mexAB-oprM (pBAD hom) | gggctaacaggaggaattaaccatgcaacgaacgccagc |
| R mexAB-oprM (pBAD hom) | gccaaaacagccaagcttcgaattcTTAtcaagcctggggatcttccttc |
| F pBAD (mexXY hom) | gcaagcctgagaattcgaagcttggctgttttg |
| R pBAD (mexXY hom) | ggatgtgcatggttaattcctcctgttagccc |
| F mexXY (pBAD hom) | gaattaaccatgcacatccaatggaccggctc |
| R mexXY (pBAD hom) | gcttcgaattctcaggcttgctccgtggg |
| R mexXY_ext (pBAD hom) | gcttcgaattctcaggcttgcgcgcc |
| F SD_ST7-oprM (pBMTL hom) | GTTTTCAGTCCAGTTACGCTGtcaagcctggggatcttccttc |
| R pBMTL (SD_ST7-oprM hom) | ggaaggaccgtttcatTTTTTTTCCTCCTTAGAGTCTGAGGCTCGTCCTGAATG |
| R oprM (pBMTL hom) | GTTTTCAGTCCAGTTACGCTGtcaagcctggggatcttccttc |
| F pBMTL (oprM hom) | gaaggaagatccccaggcttgaCAGCGTAACTGGACTGAAAAC |
